# Role of the progesterone receptor for paclitaxel resistance in primary breast cancer

**DOI:** 10.1038/sj.bjc.6603538

**Published:** 2007-01-09

**Authors:** M Schmidt, E Bremer, D Hasenclever, A Victor, M Gehrmann, E Steiner, I B Schiffer, S Gebhardt, H-A Lehr, M Mahlke, M Hermes, A Mustea, B Tanner, H Koelbl, H Pilch, J G Hengstler

**Affiliations:** 1 Department of Obstetrics & Gynecology, Medical School, University of Mainz, Mainz, Germany; 2 Coordinating Center for Clinical Trials, University of Leipzig, Leipzig, Germany; 3 Institute of Medical Biometry, Epidemiology and Information Science, University of Mainz, Mainz, Germany; 4 Bayer Health Care, Leverkusen, Germany; 5 Institute of Pathology, Johannes Gutenberg University, Mainz, Germany; 6 Center for Toxicology, University of Leipzig, Haertelstr. 16-18, 04107 Leipzig, Germany; 7 Leibniz Research Centre for Working Environment and Human Factors, University of Dortmund, Dortmund, Germany; 8 Department of Obstetrics and Gynecology, Charite, Berlin, Germany; 9 Department of Obstetrics and Gynecology, University of Leipzig, Philipp-Rosenthal-Str. 55, 04103F Leipzig, Germany

**Keywords:** paclitaxel, chemosensitivity, primary tumour cells, individualized chemotherapy, progesterone receptor

## Abstract

Paclitaxel plays an important role in the treatment of primary breast cancer. However, a substantial proportion of patients treated with paclitaxel does not appear to derive any benefit from this therapy. We performed a prospective study using tumour cells isolated from 50 primary breast carcinomas. Sensitivity of primary tumour cells to paclitaxel was determined in a clinically relevant range of concentrations (0.85–27.2 *μ*g ml^−1^ paclitaxel) using an ATP assay. Chemosensitivity data were used to study a possible association with immunohistochemically determined oestrogen and progesterone receptor (ER and PR) status, as well as histopathological parameters. Progesterone receptor (PR) mRNA expression was also determined by quantitative RT–PCR. We observed a clear association of the PR status with chemosensitivity to paclitaxel. Higher levels of immunohistochemically detected PR expression correlated with decreased chemosensitivity (*P*=0.008). Similarly, high levels of PR mRNA expression were associated with decreased paclitaxel chemosensitivity (*P*=0.007). Cells from carcinomas with T-stages 3 and 4 were less sensitive compared to stages 1 and 2 (*P*=0.013). Multiple regression analysis identified PR receptor status and T-stage as independent predictors of paclitaxel chemosensitivity, whereas the ER, N-stage, grading and age were not influential. In conclusion, *in vitro* sensitivity to paclitaxel was higher for PR-negative compared with PR-positive breast carcinoma cells. Thus, PR status should be considered as a possible factor of influence when designing new trials and chemotherapy protocols.

Taxanes like paclitaxel play an important role in the adjuvant treatment of breast cancer (Henderson *et al*, 2003). The efficacy of paclitaxel containing treatment can be further improved by dose dense protocols in which the chemotherapy is administered every 2 weeks with G-CSF support. Arguably, sequential protocols in which paclitaxel is administered not only dose dense, but also dose intensified, might prove to be even more efficient. Despite the well-documented antitumour efficacy of paclitaxel, many tumours exhibit intrinsic resistance to paclitaxel. These patients will obviously not profit from addition of paclitaxel to an anthracycline-based adjuvant chemotherapy. Identifying these patients could not only spare them an ineffective treatment, but gives the opportunity to establish a more efficient protocol for this particular subgroup of paclitaxel-resistant patients. In the adjuvant setting, Henderson *et al* (2003) have shown that especially patients whose tumours were oestrogen receptor (ER) negative derived most of the benefit from adding paclitaxel to an anthracyclin-based regimen. However, it is not entirely clear whether in this setting the prognostic impact of the hormone receptor status and the effect of adjuvant treatment with tamoxifen might thus offset a potential predictive effect of the hormone receptor status for paclitaxel chemosensitivity.

A large number of studies have been performed to identify predictive markers for chemosensitivity and/or prognosis of carcinomas (Hengstler *et al*, 1992, 2001; Micke *et al*, 2001, 2003; Brenner *et al*, 2002; Hast *et al*, 2002; Schiffer *et al*, 2003; Steiner *et al*, 2003; Mohrmann *et al*, 2005). Resistance to paclitaxel can be induced by decreased expression of the spindle assembly checkpoint genes Mad2 and BubR1 (Sudo *et al*, 2004). In contrast, low expression of the microtubule-associated protein tau was associated with high sensitivity to paclitaxel (Rouzier *et al*, 2005). High expression of beta-tubulin III has been reported to predict progression after paclitaxel chemotherapy (Paradiso *et al*, 2005). Thus, genes involved in spindle assembly have a high probability to be involved in paclitaxel resistance. This corresponds to the mechanism of action, as paclitaxel stabilises microtubules. Resistance to paclitaxel (Duan *et al*, 2005) and docetaxel (Chang *et al*, 2003) has been reported to be associated with specific patterns of gene expression. Interestingly, chemosensitivity of primary tumour cells could be increased by inhibition of P-glycoprotein (Di Nicolantonio *et al*, 2004). These examples demonstrate that many factors that might predict paclitaxel chemosensitivity of breast cancer patients have been discovered. If validated, these factors could help to reduce unnecessary treatment for women with breast cancer or help to identify sensitive subpopulations. An important milestone in translation of preclinical concepts to clinical application is the demonstration that new predictive factors are independent from the classical histopathological parameters. However, the influence of histopathological factors, such as ER and PR status, T-stage, N-stage, grading and patient's age on paclitaxel chemosensitivity of the breast carcinoma cells is still not clear. To clarify this controversial issue, we performed a prospective study in 53 consecutive breast cancer patients using an *in vitro* chemosensitivity assay (ATP-TCA, Andreotti *et al*, 1995; Kurbacher *et al*, 1996; Hengstler *et al*, 1999b) with primary tumour cells isolated from resected breast cancer tissue. Here, we report for the first time that PR status and tumour stage are independent predictors of paclitaxel chemosensitivity in primary breast cancer cells.

## MATERIALS AND METHODS

### Patients

We used fresh breast cancer tissue specimens of consecutive patients who underwent surgery for primary breast cancer at the Department of Obstetrics & Gynecology, University of Mainz, Medical School during October 2002–September 2003. In this period of time, 155 patients had surgery for primary breast cancer. In 53 of these patients, a sufficient amount of fresh breast cancer tissue (at least 0.5 cm^3^) allowed for *in vitro* chemosensitivity testing. Patient characteristics are shown in Table 1.

### *In vitro* chemosensitivity assay (ATP-TCA)

The chemosensitivity test was performed with primary tumour cells that have been isolated from tumour tissue immediately after resection. A commercially available kit (TCA-100; DCS, Innovative Diagnostic Systeme, Hamburg, Germany) was used to assess chemosensitivity according to the manufacturer's instructions. Briefly, tumour specimens were dispersed using sterile scalpels. Subsequently, small tissue fragments were enzymatically dissociated. After filtration and ficoll-hypaque density centrifugation, the quality and viability of the single cell suspension were assessed by trypan blue dye exclusion and cytological examination. Adding serum-free Complete Assay Medium (CAM; DCS, Innovative Diagnostic Systeme, Hamburg, Germany) cell suspensions were adjusted to a final concentration of 1–2 × 10^5^ viable cells per ml. Assays for paclitaxel chemosensitivity were performed in 96-well polypropylene microtitre plates. Test drug concentrations were administered in triplicate in six different concentrations: 0.85, 1.7, 3.4, 6.8 and 13.6, 27.2 *μ*g ml^−1^ paclitaxel. Two controls were included for analysis of each tumour, a negative control with complete assay medium (M0) and a positive control containing Maximum ATP Inhibitor (MI, Innovative Diagnostic Systeme, Hamburg, Germany) instead of paclitaxel. Subsequently, 100 *μ*l of single cell suspension corresponding to 15 000 cells were added to each well. These cultures were then incubated at 37°C and 95% humidity in 5% CO_2_ atmosphere. After 6 days of incubation and a cytological analysis of untreated controls, intracellular ATP was extracted and stabilised by addition of 50 *μ*l tumour cell extraction reagent (TCER; DCS, Innovative Diagnostic Systeme, Hamburg, Germany) to the remaining wells of the culture plates. A 50 *μ*l aliquot of each well was then transferred into a luminometer (LB-953 luminometer, Berthold, Wildbad, Germany). After pipetting 50 *μ*l of luciferin–luciferase reagent (Lu-Lu; DCS, Innovative Diagnostic Systeme, Hamburg, Germany) to each cell extract, the ATP concentration was measured. An ATP standard curve was included into all assays. Three independent incubations with primary tumour cells of each patient were performed with all paclitaxel concentrations (0.85, 1.7, 3.4, 6.8 and 13.6, 27.2 *μ*g ml^−1^), and six independent incubations with the culture medium controls. Median values for each concentration were used to calculate the area under the curve (AUC). From a total of 53 tested breast cancer specimens, three showed a too low ATP content of the culture medium controls after a culture time of 6 days probably owing to a too low viability of the isolated tumour cells. Therefore, the overall evaluability rate was 94%.

### Immunohistochemistry

Serial sections of formalin-fixed slices were stained with either monoclonal ER antibodies (clone 1D5, Dako, Glostrup, Denmark) or monoclonal PR antibodies (clone PgR 636, Dako, Glostrup, Denmark), as described (Steiner *et al*, 2003). The immunohistochemical evaluation was performed by one of the authors (MS) trained in histological and immunohistochemical diagnostics, unaware of the ATP-TCA data.

### Quantitative RT–PCR for PRs A and B

Total RNA was isolated from 5 *μ*M sections of formalin-fixed, paraffin-embedded tumour tissues after histopathological confirmation of a tumour cell content of at least 70%. Reverse transriptase–polymerase chain reaction (RT–PCR) was performed as described Hengstler *et al* (1999a). Primers and probes were designed for both the exclusive detection of the PR-B isoform as well as the simultaneous detection of the PR-A and PR-B isoforms using the Primer Express software (Applied Biosystems, Foster City, CA, USA). Sequences of probe, forward and reverse primer for (i) PGR (isoform B) and (ii) PGR (isoform A+B) were:

(i) Probe: 5′ TCGCAGCAGGAGAAACTTGAAAGCATTC 3′
  Forward: 5′ TCAAGAGGAGCAGGACATGTTG 3′
  Reverse: 5′ TTCTCTCCCTTATGAGTTCCATAAAAG 3′
(ii) Probe: 5′ TTGATAGAAACGCTGTGAGCTCGA 3′
  Forward: 5′ AGCTCATCAAGGCAATTGGTTT 3′
  Reverse: 5′ ACAAGATCATGCAAGTTATCAAGAAGTT 3′

To standardise the amount of sample RNA, *GAPDH* was selected as a reference gene. Primer and probes were obtained from Eurogentec s.a. (Liege, Belgium) and the sequences are shown above.

### Statistical analysis

The Statistical Package for Social Science (SPSS 12.0, Inc., Chicago, IL, USA) was used for statistical analysis. Differences between AUC values between two groups were evaluated using the *t*-test. Local significance was considered as two-sided *P*<0.05. Linear regression analysis was used to model dependence of chemosensitivity (AUC) on multiple covariates.

## RESULTS

### Paclitaxel chemosensitivity is associated with PR status and tumour stage

**Figure 1:**
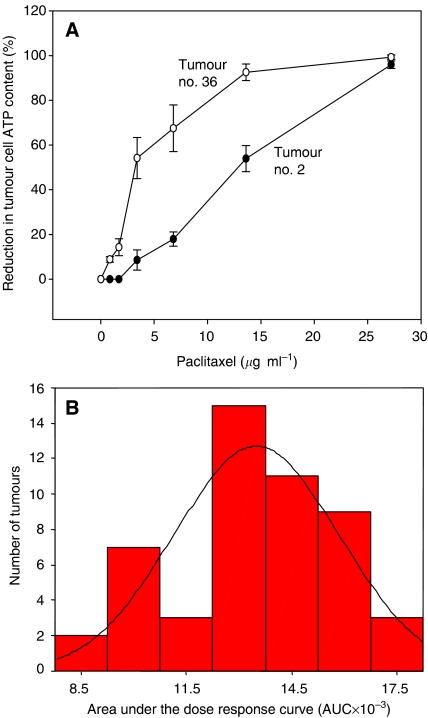
(**A**) Representative results of the *in vitro* chemosensitivity assay. Tumour no. 36 is relatively sensitive (AUC: 16.6) in contrast to tumour no. 2 (AUC: 9.5). A concentration of 3.7 *μ*g ml^−1^ paclitaxel corresponds to the peak plasma concentration reached in patients. All data points are mean values and standard deviations from three independent incubations. (**B**) Frequency distribution of the AUCs of all 50 tumours.

**Figure 2:**
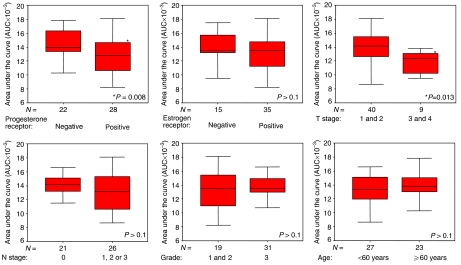
Association between chemosensitivity to paclitaxel (expressed as AUC) and histopathological parameters. Only the progesterone receptor (PR) status (*P*=0.008) and T-stage (*P*=0.013) correlated with chemosensitivity.

For evaluation of paclitaxel chemosensitivity primary tumour cells isolated from 50 breast cancer patients were analysed (baseline characteristics: Table 1). The area under the dose–response curve (AUC) was used as a measure for chemosensitivity. Representative examples of a relatively sensitive and a relatively resistant tumour as well as the frequency distribution of all AUCs are shown in Figure 1. Next we analysed whether the AUC corresponds to the progesterone and ER status (positive *vs* negative), as well as to T-stage (stages 3 and 4 *vs* 1 and 2), N-stage (stages 1, 2, 3 *vs* 0), grading (grade 3 *vs* 1 and 2) and age (older *vs* younger than 60 years) (Figure 2). A clear association was observed between PR status and the AUC (*P*=0.008), whereby a positive receptor status was associated with decreased chemosensitivity (Figure 2). Similarly, T-stage was associated with chemosensitivity to paclitaxel. T-stage 3 and 4 carcinomas had lower AUCs compared with T-stage 1 and 2 (*P*=0.013). In contrast, ER status, N-stage, grading and age were not associated with chemosensitivity to paclitaxel (Figure 2). Similar results were obtained when the IC_90_ was applied for evaluation instead of the AUC (data not shown).

### Multiple regression analysis identifies PR status and T-stage as independent predictors of paclitaxel chemosensitivity

To analyse which of the individual parameters shown in Figure 2 are independent predictors of paclitaxel chemosensitivity, we performed a multiple linear regression analysis. Using a step down approach, initially the PR status, T-stage, N-stage, grading and age were included. The final model included only PR status (positive *vs* negative, *P*=0.015) and T-stage (stages 3 and 4 *vs* 1 and 2, *P*=0.021) as independent parameters of influence (*P*=0.014).

### PR-A and -B isoforms both are associated with paclitaxel chemosensitivity

**Figure 3:**
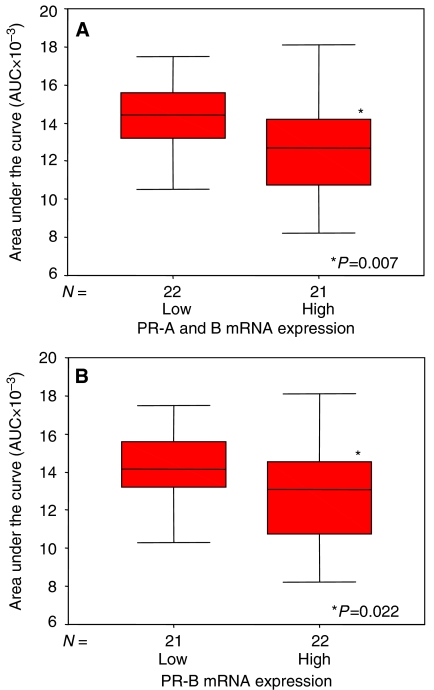
Association between paclitaxel chemosensitivity and PR mRNA expression. High expression of PR-A+B (**A**) and PR-B (**B**) mRNA expression is associated with decreased sensitivity (AUC) of the breast cancer cells to paclitaxel.

Next, we studied whether the correlation between paclitaxel chemosensitivity and PR status could be confirmed on the mRNA level. In accordance with previous studies we performed quantitative RT–PCR using a primer pair specific for PR-B and another primer pair amplifying both, PR-A and -B. High-quality RNA could be isolated from only 46 of the 50 patients. mRNA expression of PR-A and -B as well as of PR-B alone were dichotomised using the respective medians as cutpoints. Both, PR-A and -B as well as PR-B alone were associated with chemosensitivity to paclitaxel (*P*=0.007 and *P*=0.022, respectively), whereby tumours with relatively high levels of receptor expression appeared less sensitive to paclitaxel compared with carcinomas with low levels of the PR (Figure 3A and B). Thus, RT–PCR analysis (Figure 3) confirmed the correlation between the immunohistochemically (Figure 2A) determined PR expression and paclitaxel chemosensitivity. The difference in chemosensitivity seems to be slightly higher if PR-A and -B expression (Figure 3A) was considered compared to PR-B alone (Figure 3B), suggesting that PR-A might be more relevant than PR-B with respect to chemosensitivity. However, owing to the relatively small difference and owing to the fact that we did not specifically quantify PR-A, conclusions as to possible differences between PR isoforms should be treated with caution. In conclusion, the association of the PR with paclitaxel chemosensitivity could be confirmed on the mRNA level.

### Influence of PR status and T-stage on paclitaxel dose—response curves

**Figure 4:**
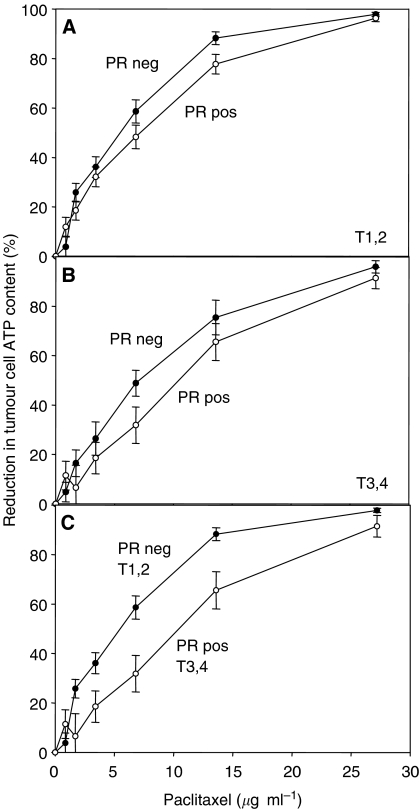
Sensitivity of primary breast cancer cells from 50 patients to paclitaxel. (**A**) Influence of the progesterone receptor (PR) status in primary tumour cells from patients with T-stage 1 or 2. (**B**) Influence of the PR status in primary tumour cells from patients with T-stage 3 and 4. (**C**) Combined influence of PR status and T-stage: patients with PR-negative tumours, T-stage 1 or 2 *vs* patients with PR-positive tumours, T-stage 3 or 4. The difference between the two groups was significant (*P*<0.01) for paclitaxel concentrations of 1.7, 3.4, 6.8 and 13.6 *μ*g ml^−1^. In contrast, no significant difference was obtained for the highest concentration of 27.2 *μ*g ml^−1^ paclitaxel.

To be able to quantitatively assess the association of PR status with paclitaxel chemosensitivity we separately analysed tumour cells from T-stage 1 or 2 as well as T-stage 3 or 4 patients. In the group of T-stage 1 or 2 tumours a higher sensitivity was observed for the PR-negative cells (Figure 4A). The difference in the AUC between PR-negative and -positive cells in T-stage 1 or 2 was significant (*P*=0.027). A similar association between PR and sensitivity to paclitaxel was seen in cells from T-stage 3 or 4 tumours (Figure 4B, *P*=0.049). The largest difference was obtained between PR-negative/T-stage 1 or 2 *vs* PR-positive/T-stage 3 or 4 tumours (Figure 4C, *P*=0.001). In the concentration range between 1.7 and 3.4 *μ*g ml^−1^ approximately two-fold higher concentrations of paclitaxel are required for PR positive/T-stage 3 or 4 tumours to achieve a toxicity similar to that as for PR-negative/T-stage 1 or 2 tumours.

## DISCUSSION

Selecting patients with primary breast cancer for the most appropriate adjuvant systemic treatment is of great importance. The only currently accepted and reliable predictive factor for clinical decision making in the adjuvant setting is the hormone receptor status (Goldhirsch *et al*, 2005). Besides hormone receptors, there is no generally accepted factor for the prediction of response to chemotherapy in breast cancer. HER-2/neu was retrospectively investigated as a predictive factor for an anthracycline-based therapy in comparison with CMF (Paik *et al*, 1998; Moliterni *et al*, 2003). However, owing to inconsistent results, HER-2/neu is currently not recommended in this setting (Goldhirsch *et al*, 2005). In an explorative subgroup analysis of the randomised clinical trial CALGB 9344, Henderson *et al* (2003) observed evidence that the ER status might be predictive for a benefit of paclitaxel added to a standard anthracyclin-based regimen. Patients with ER-positive tumours had a greater benefit from the addition of paclitaxel to an anthracycline-based therapy than patients with ER-negative tumours. However, based on this finding, it is unclear whether this is truly predictive for paclitaxel response or whether the well-known prognostic effects of the hormone receptor status itself come into play. Other clinical trials also investigated a possible interaction between the ER status and paclitaxel (Mamounas *et al*, 2005) or docetaxel (Nabholtz *et al*, 2002). However, these trials failed to demonstrate any interaction between clinical outcome and ER expression.

For the above mentioned reasons, the adjuvant setting is not ideal to investigate the predictive effect of a biomarker. A design which is much more appropriate for this particular purpose was used in the National Surgical Adjuvant Breast and Bowel Project Protocol (NSABP) B-27. In this clinical trial, patients with primary breast cancer were treated with primary systemic chemotherapy either with or without docetaxel. These authors again failed to confirm any association between the ER status and the benefit from the use of a taxane containing regimen. However, owing to the concurrent administration of tamoxifen and chemotherapy, the potential predictive effect of the ER for chemotherapy response might be obscured.

In the present study, we excluded potential prognostic effects as well as interference with other systemic therapies by using a well-established *in vitro* assay (ATP-TCA) for primary tumour cells (Andreotti *et al*, 1995; Cree *et al*, 1996; Kurbacher *et al*, 1996; Hengstler *et al*, 1999b; Konecny *et al*, 2000) in a consecutive series of 53 primary breast cancers. The ATP-TCA has been shown to allow a prediction of clinical results. The ATP-TCA-directed chemotherapy has improved clinical outcome in several studies (Andreotti *et al*, 1995; Cree *et al*, 1996; Kurbacher *et al*, 1996; Konecny *et al*, 2000). It should be considered that our ATP assay measures chemosensitivity on a cellular level, which is only one of several parameters relevant for clinical outcome. Other relevant parameters, such as local pharmacokinetics, tumour vascularisation, oxygen supply or immune response will not be considered by our *in vitro* technique. Nevertheless, the applied assay determines whether tumour cells *ex vivo* dispose of mechanisms protecting them from paclitaxel toxicity *in vitro*. This is likely to be one of several parameters contributing to chemosensitivity of a tumour *in vivo*. Our assay evaluability rate of 94% is well in line with the literature (Cree *et al*, 1996; Kurbacher *et al*, 1996). The present study was designed to study a possible association of ER and PR status with chemosensitivity of breast cancer cells in the ATP-TCA and to analyse whether a possible association is independent from the classical clinical factors, such as T-stage, N-stage, grading and age.

In the present prospective study, we observed a clear association between the PR status and chemosensitivity to paclitaxel. Higher levels of immunohistochemically detected PR expression correlated with decreased chemosensitivity. To confirm this observation by a second, independent technique, we measured PR expression also by quantitative RT–PCR. Progesterone exerts its effects through two nuclear receptors, PR-A and PR-B, which are encoded by a single gene, under the regulation of two distinct promoters (Kastner *et al*, 1990). The two receptor proteins are identical except that PR-B contains an additional 164 amino acids at its N-terminal end that are absent from PR-A. These two PR isoforms can be distinguished by immunoblot analysis, but not by ligand-binding assays or by immunohistochemistry. In accordance with previous studies, we used two pairs of primers amplifying either a fragment of the identical part (PR-A and -B) as well as a fragment specific for PR-B. Similar to the result obtained by immunohistochemistry, PR mRNA expression also correlated with chemosensitivity. A high correlation of the two isoforms with each other and also with total PR has been reported, indicating that virtually every PR-positive breast tumour expresses at least some level of PR-A and -B (Hopp *et al*, 2004). In our study, both, PR-A and -B and PR-B mRNA expression correlated with decreased chemosensitivity to paclitaxel.

We did not address the question of the molecular mechanisms responsible for PR-mediated chemoresistance in the present study. However, previous studies have demonstrated an up regulation of the antiapoptotic gene BCL-XL in breast cancer cells as a consequence of PR-A expression (Richer *et al*, 2002). This upregulation could lead to resistance to apoptosis. As apoptosis is a major factor for the cytotoxic effects exerted by paclitaxel (Woods *et al*, 1995; Milross *et al*, 1996), it might explain why PR-positive tumours were more resistant to paclitaxel compared with PR-negative carcinomas. However, besides BCL-XL, at least 93 further genes have been shown to be PR-A and/ or PR-B dependent in breast cancer cells (Richer *et al*, 2002). Therefore, it can not be excluded that PR-mediated chemoresistance is multifactorial, whereby BCL-XL may represent only one of several factors. Nevertheless, the antiapoptotic influence of the PR is well documented. For instance, serum depletion-induced apoptosis was inhibited by progesterone treatment (Ory *et al*, 2001) and also radiation-induced apoptosis could be antagonised via PR (Vares *et al*, 2004) in breast cancer cell lines.

Using multiple regression analysis we demonstrated that an association of the PR with paclitaxel chemosensitivity is independent from classical clinical prognostic factors. However, a second factor was found to be influential, namely the T-stage. To be able to quantitatively assess the association of PR status with T-stage, we compared the dose–effect curves of four subgroups tumours: (i) PR negative and T-stage 1 or 2, (ii) PR negative and T-stage 3 or 4, (iii) PR positive and T-stage 1 or 2 and (iv) PR positive and T-stage 3 or 4. Tumours of PR-negative/T-stage 1 or 2 tumours were clearly more sensitive than PR-positive/T-stage 3 or 4 tumours. The concentrations used in our *in vitro* study are 0, 0.85, 1.7, 3.4, 6.8 and 13.6, 27.2 *μ*g ml^−1^ paclitaxel. The choice of these concentrations is based on pharmacokinetic data of a phase 3 randomised study following 3- and 24-h infusions of paclitaxel at dose levels of 135 and 175 mg m^−2^. Maximum plasma concentrations were 3.65 *μ*g ml^−1^ for 3 h infusion of 175 mg m^−2^ paclitaxel. Therefore, a clinically relevant dose range has been chosen in the present study. In this range of clinically relevant concentrations, approximately two-fold higher concentrations are required for PR-positive/T-stage 3 or 4 tumours to achieve a similar toxicity compared to PR-negative/T-stage 1 or 2 tumours. It is likely that both PR and T-stage are only two of many factors influencing paclitaxel sensitivity of breast cancer. Nevertheless, the results of this study demonstrate that PR and T-stage should be considered as influential parameters in studies aimed at identifying new factors that might predict paclitaxel resistance.

In conclusion, our prospective study in primary breast cancer highlights the importance of the PR for the chemosensitivity to paclitaxel. Clearly, these *ex vivo* data should be taken as hypothesis-generating results which have to be confirmed in larger clinical trials.

## Figures and Tables

**Table 1:** Baseline characteristics of the study population

	**Number evaluated (*n*=50)**	**%**	**Not evaluable**
Age (years)	59.2±13.5^a^		
			
*T-stage*			1
T1	20	40.8	
T2	20	40.8	
T3	3	6.1	
T4	6	12.2	
			
*N-stage*			3
N0	20	42.6	
N1	20	42.6	
N2	2	4.3	
N3	5	10.6	
			
*Grading*			0
Grade 1	7	14.0	
Grade 2	12	24.0	
Grade 2	31	62.0	
			
*Histological type*			1
Invasive ductal carcinoma	41	83.7	
Other types	8	16.3	
			
*Estrogen receptor* b			0
Positive	35	70.0	
Negative	15	30.0	
			
*Progesterone receptor* b			0
Positive	28	56.0	
Negative	22	44.0	

a Mean±s.d.

b Immunohistochemically determined.
